# Computerized-adaptive testing versus short forms for pediatric inflammatory bowel disease patient-reported outcome assessment

**DOI:** 10.1017/cts.2023.526

**Published:** 2023-04-14

**Authors:** Erica J. Brenner, Li Lin, Kirsten M. Bahnson, Millie D. Long, Wenli Chen, Michael D. Kappelman, Bryce B. Reeve

**Affiliations:** 1 University of North Carolina, Department of Pediatrics, Division of Pediatric Gastroenterology, Chapel Hill, NC, USA; 2 Duke University School of Medicine, Department of Population Health Sciences, Durham, NC, USA; 3 University of North Carolina, Department of Medicine, Division of Gastroenterology, Chapel Hill, NC, USA

**Keywords:** Patient-reported outcomes, computerized-adaptive testing, short forms, inflammatory bowel disease, Crohn’s disease, ulcerative colitis

## Abstract

**Introduction:**

Computerized-adaptive testing (CAT) may increase reliability or reduce respondent burden for assessing patient-reported outcomes compared with static short forms (SFs). We compared CAT versus SF administration of the Patient-Reported Outcomes Measurement Information System® (PROMIS®) Pediatric measures in pediatric inflammatory bowel disease (IBD).

**Methods:**

Participants completed 4-item CAT, 5- or 6-item CAT, and 4-item SF versions of the PROMIS Pediatric measures. We compared average T-scores, intra-class correlations (ICCs), floor and ceiling effects, and standard error of measurement (SEM) across forms, along with mean effect sizes between active versus quiescent IBD disease activity groups.

**Results:**

Average PROMIS T-scores across forms were <3 points (minimally important difference) of each other. All forms correlated highly with each other (ICCs ≥0.90) and had similar ceiling effects, but the CAT-5/6 had lower floor effects. The CAT-5/6 had lower SEM than the CAT-4 and SF-4, and the CAT-4 had a lower SEM than the SF-4. Mean effect sizes were similar across forms when contrasting disease activity groups.

**Conclusions:**

The CAT and SF forms produced similar score results, but the CAT had better precision and lower floor effects. Researchers should consider PROMIS pediatric CAT if they anticipate that their sample will skew toward symptom extremes.

## Introduction

Over the past two decades, the healthcare research field has increasingly recognized the importance of capturing symptoms directly from patients. This movement to incorporate patient-reported outcomes (PROs) in chronic conditions such as inflammatory bowel disease (IBD) is supported by studies demonstrating discordance between patient and provider assessment of patient symptoms and functioning [1,2]. A better understanding of symptom burden and functioning is critical to adequately treating IBD and other chronic conditions in which disease activity relapses and remits throughout the lifespan and associates significantly with psychosocial health. Symptoms such as pain and fatigue greatly impact patients’ daily functioning (e.g., going to school) and quality of life [3,4]. Thus, PROs are recommended to comprehensively assess patient functioning and symptom burden and to evaluate treatment effectiveness. The best way to administer PRO measures, however, is a topic of ongoing discussion.

In 2004, the National Institutes of Health (NIH) sponsored the creation of the Patient-Reported Outcomes Measurement Information System® (PROMIS®), a collection of person-centered measures designed for use in clinical trials and clinical practice settings [5]. The PROMIS Pediatric measures were developed specifically for child respondents [6], and studies have found evidence for their reliability and validity in the general population and in children with chronic conditions such as pediatric IBD [6–12]. The PROMIS Pediatric measures, like other PRO measures, assess each symptom or functioning domain with multiple items as part of a scale. Although other tools exist to assess patient functioning, including IBD-specific disease burden indices, like the TUMMY-UC scoring system [13], general quality of life indices, such as the IMPACT-III [14], and clinical screening tools such as the GAD-7 [15] or PHQ-9 [16], the PROMIS Pediatric measures are unique in that they provide investigators great flexibility in selecting only the relevant health-related quality of life domains for their population and/or study goals. In addition, the PROMIS system allows investigators to select the length of the short form (e.g. 4-items, 6-items, or 8-items per domain) or select computerized-adaptive testing (CAT) based on the needed precision of measurement [6]. Further, the PROMIS Pediatric measures were designed following best practices in both qualitative and quantitative measures and validated in multiple pediatric populations.

Most commonly, all patients who complete the PRO measures answer the same set of questions on each symptom to allow comparison between subgroups (e.g., intervention vs control arm) or modeling of symptom experiences over time in the same individuals (e.g., baseline and post-intervention). For purposes of this paper, we will call these *static short forms (SFs)* to represent how each item in the PRO scale is always administered [17].

For PRO measures to function optimally in chronic disease research, they must comprehensively evaluate the burden of disease including symptom range and effect on daily functioning, detect change over time and between subgroups, and minimize respondent burden [18]. These goals present challenges for static SFs. First, capturing a broad range of symptom, function, and QOL domains, while ideal, can result in long questionnaires that patients may tire or grow bored of prior to completion [18,19]. Consequently, patients may either quit the survey early, resulting in missing data, or provide careless or rushed answers, resulting in inaccurate results [18,19]. If researchers try to circumvent this problem by administering only one or a few questions per domain, they often end up with imprecise PRO measurements that cannot detect meaningful change over time or differences between subgroups [20]. This issue holds special relevance for patients with very severe symptom burden and who often score at the ceiling of symptom measures (representing severe symptom impact), as the short PRO measure focuses more on mild to moderate symptom experiences where a majority of the sample is typically located [21].

Computerized-adaptive testing (CAT) has been touted as a potential solution to these challenges, as it may reduce the length of a PRO measure compared to a static SF without sacrificing precision, or, for a given same scale length (e.g., 4-items), achieve better reliability than a static SF, especially at the floor or ceiling of the measure [17,22]. In brief, CAT individually tailors the PRO assessment by selecting the most informative set of questions from an *item bank* based on how each patient answers questions in the same PRO domain [17,22]. Thus, a CAT-based PRO assessment may capture a broader range of symptom and function domains than the SF with short and reliable measures. However, the CAT requires costly software programming and must be administered electronically, meaning participants must have access to computer equipment and/or tablets. Thus, researchers must weigh practical considerations along with the characteristics of the particular study population and the study aims to decide between the CAT and SF assessment. The PROMIS Pediatric item banks offer access to both static SFs and CAT-based measures.

The objective of this study is to characterize the tradeoffs of using a static SF versus CAT PROs assessments by comparing CAT and SF administration of the PROMIS Pediatric measures in a cohort of children and adolescents with IBD. The study results may inform pediatric clinical trialists who are deciding whether to use the static SFs or invest in a CAT-based system to administer PROMIS measures. We sought to determine the degree to which CAT and SF scores correlate and to compare the performance of CAT versus SF, particularly regarding score distributions (i.e., floor and ceiling effects), precision, and discriminatory power. We anticipated that the CAT and SFs would correlate strongly, and that the CAT would be more efficient, have fewer floor and ceiling effects, and produce a more precise estimate.

## Materials and Methods

This prospective study cohort was drawn from IBD Partners Kids & Teens, a web-based platform established in 2013. Pediatric patients with self-reported IBD and their parents across the United States were invited to participate using website recruitment advertisements, emails, and flyers. Parents were provided with information about the study and given the ability to provide informed consent. Assent was obtained from the pediatric participants. Parents and patients were then asked to fill out surveys, including the PROMIS Pediatric measures and various questions on disease and demographic characteristics. Patients and parents were asked to complete surveys at baseline and then every 1.5–6 months. IBD Partners Kids & Teens was inspired the adult version, IBD Partners, which is a web-based cohort started in 2011 (methodology described elsewhere [23]). We included only patients ages 9–17 years from this cohort, as this group completed self-report forms, while parents completed outcome questions for participants younger than 9 years of age [9]. Patients with Crohn’s disease, ulcerative colitis, and IBD unspecified were included in the study population.

### Measures

The NIH-sponsored PROMIS Pediatric domains are a set of PROs that measure functioning and symptoms directly from the pediatric patient’s point of view [6]. The present study included PROMIS Pediatric domains of anxiety, depressive symptoms, fatigue, and pain interference. Higher scores represent more severe symptoms, and a minimally important difference (MID) was determined to be 3 points [24]. Because all questions on the static SF and the CAT are selected from the same PROMIS Pediatric item bank, their scores can be compared across the assessment modes. The PROMIS 4-item SF used in this study is available from the PROMIS website (HealthMeasures.net) and items were selected as they have discrimination ability across the continuum of the symptom being assessed. The PROMIS CAT algorithm selects questions based on their discrimination ability as well as selecting items that further probe the respondent’s likely symptom level, estimated based on their answers to previous items administered by the CAT. The CAT algorithm, Adaptest®, was provided by Vector Psychometric Group. The CAT algorithm starts by assuming the child has a score at the calibration sample mean PROMIS T-score of 50 and selects the most discriminating item. The CAT algorithm stopping rule was set up based on scale length (minimum 5 and maximum 6 items) and stopped at 5 items if the standard error of measurement (SEM) is less than 0.40 standardized units (4 points on the PROMIS T-score metric). For this study, we report on both the full administration of the CAT with five or six questions (CAT-5/6) and the CAT with only the first 4 answered questions (CAT-4). The CAT-4 allows us to examine statistical properties measured when holding the number of administered questions the same as the 4-item static SF (SF-4). For this study, participants completed the CAT assessment first until the stopping rule was achieved, then completed any additional questions from the 4-item SF that were not already administered as part of the CAT. Although IBD Partners Kids & Teens is a longitudinal observational study, this study only used one of each participant’s answers to the PROMIS Pediatric SFs and CAT at a single timepoint. In some cases, it was a child’s first assessment point that had complete data on each measure, and in other cases, it was the second assessment point that had complete data. However, we performed a sensitivity analysis that included all the complete data from participants (i.e. complete data from subsequent surveys), using mixed modeling to account for within-person clustering. Since the number of participants with completed subsequent data was small and results did not change, we only included data from their first completed surveys.

### Analyses

Consistent with PROMIS scoring algorithms, response pattern scoring on the SF-4, CAT-4, and CAT 5/6 were estimated using expected a posteriori (EAP) estimation, with a linear transformation to place the scores on a T-score metric. We tested differences in PROMIS Pediatric T-scores between the forms (SF-4, CAT-4, CAT-5/6) with the dependent samples *t*-test (i.e. paired *t*-test) to examine if their scores differed statistically based on the type of form. We hypothesized the mean scores would not be statistically different. Association of scores from the different forms was estimated with intra-class correlations (ICCs) for absolute agreement by using mixed linear models [25]. We expect the SF and CAT scores to be highly correlated, as they are derived from the same item bank and measure the same construct; in some cases, both versions being compared included the same item(s). Descriptive statistics are used to summarize floor and ceiling effects among the three forms. Supplementary Table 1 shows how the minimum and maximum scores for each domain (used to determine floor and ceiling effects) vary by CAT and SF administration.

We estimated SEM differences among the three forms (SF-4, CAT-4, CAT-5/6) with a dependent samples *t*-test in order to test the relative precision of the different forms. In item response theory, the SEM varies conditionally on the level of the construct (i.e., PROMIS domain) being measured and is inversely related to reliability (precision). The magnitude of SEM depends on the item parameters and the number of administered items. We expected the SEM to be lowest for the CAT-5/6 and the SF-4 to have the highest SEM. Graphically, we present SEM plots for each form for each PROMIS Pediatric symptom domain. To test this, we first performed an ANOVA for an omnibus test for each PROMIS Pediatric domain to see if the average SEMs are different among the three forms. If we found there were statistically significant differences, then we ran post-hoc pairwise comparisons between each form by testing if the difference in means is different from zero.

We examined if the forms showed statistically different effect sizes when contrasting children with active IBD disease versus children in remission. Children with Crohn’s disease were considered to be in remission if their short Crohn’s Disease Activity Index (sCDAI) was < 150 [26], and children with ulcerative colitis were considered to be in remission if their Pediatric Ulcerative Colitis Activity Index (PUCAI) was < 10 [27,28]. Effect sizes were calculated by subtracting the means and standardizing them based on the pooled standard deviations (SDs) from active disease and remission groups. Statistically significantly different effect sizes were determined if their 95% confidence intervals (CIs) did not overlap.

All tests were two-tailed, *α* = 0.05. SAS software version 9.4 (copyright © 2016 by SAS Institute Inc., Cary, North Carolina, United States of America) was used for all analyses.

### Ethical Considerations

The IBD Partners Kids & Teens protocol and the Pediatric Patient Reported Outcomes in Chronic diseases (PEPR) Coordinating Center were reviewed and approved by the Institutional Review Boards (IRB) of the University of North Carolina at Chapel Hill and Duke University School of Medicine, respectively. Electronic consent from pediatric participant’s guardians and assent from pediatric participants were obtained at the time of cohort enrollment.

## Results

### Participant Characteristics

We evaluated 143 children and adolescents with IBD, of which 73% had Crohn’s disease, 49% were female, and the average age was 14.0 years (SD 2.2 years). Participants from 34 states across the United States filled out surveys. Table 1 lists demographic and disease characteristics for the cohort.

**Table 1.** Participant characteristics

Demographics^a^	Entire study cohort (*N* = 143)
Age	
mean (SD)	14.0 (2.2)
median (25^th^ percentile; 75^th^ percentile)	14.0 (13.0–16.0)
Sex, n (%)	
Female	70 (49.0%)
Male	73 (51.0%)
Race^b^, n (%)	
African American	3 (2.1)
Asian	3 (2.1)
Multiracial	4 (2.8)
White	132 (93.0)
Other	0 (0)
Ethnicity, n (%)	
Hispanic	8 (5.6)
Non-Hispanic	134 (94.4)
Highest parental education, n (%) (mother, father)	
Less than 12^th^ grade	3 (2.1); 2 (1.4)
12^th^ grade	8 (5.6); 10 (7.0)
Some college	10 (7.0); 23 (16.2)
College	59 (41.5); 51 (35.9)
Graduate school	62 (43.7); 51 (35.9)
Unknown	0 (0); 1 (0.7)
Type of IBD, n (%)	
Crohn’s disease	105 (73.4%)
Ulcerative colitis	31 (22.7%)
IBD unspecified	7 (4.9%)
Disease characteristics	
Age at IBD diagnosis^c^, mean (SD)	8.8 (3.7)
Years since IBD diagnosis, mean (SD)	5.3 (3.3)

a Percentages do not include missing values. For all demographic characteristics, less than 4% of data was missing for each category.

b Participants could choose more than one race.

c Value was set to 0.5 years for all respondents who answered “less than one year” for age at IBD diagnosis.

SD, standard deviation; IQ range, Interquartile range; IBD, inflammatory bowel disease.

### CAT and SF Characteristics

For the CAT-5/6, the majority of respondents were given six questions (76% for anxiety, 52% for depressive symptoms, 83% for fatigue, and 63% for pain interference). The number of items in common between the CAT and the SF is shown in Supplementary Table 2. As expected, there was substantial overlap, with most CAT forms having 1–3 items in common with the SF for each PROMIS Pediatric domain. Consistent across the forms, the median time for a child to complete an item ranged between 5 and 6.25 seconds.

### Score Differences Among the Forms

Table 2 presents the PROMIS Pediatric T-score means for each form by symptom domain. The PROMIS Pediatric mean scores were similar across the SF and the CATs for anxiety. For depressive symptoms, there was a statistically significant difference between the SF-4 and CAT-4. For pain interference, there was a statistically significant difference between the CAT-4 and the CAT-5/6. For fatigue, all forms had average scores that demonstrated a statistically significant difference from each other. None of the mean score differences reported in Table 2 exceeded the MID of 3 points.

**Table 2.** Mean PROMIS pediatric T-scores for short form (SF) and computerized-adaptive testing (CAT) assessments

	Individual forms			Difference between forms		
	SF–4	CAT–4	CAT–5/6	SF-4 – CAT-4	SF-4 – CAT-5/6	CAT-4 – CAT-5/6
	mean (95% CI; SD)	mean (95% CI; SD)	mean (95% CI; SD)	mean (95% CI; SD)	mean (95% CI; SD)	mean (95% CI; SD)
Anxiety	46.02 (44.40–47.65; 9.83)	45.99 (44.37–47.61; 9.67)	45.73 (44.06–47.39; 9.74)	0.14 (−0.58–0.85; 4.28)	0.53 (−0.09–1.15; 3.67)	0.24 (−0.09–0.58; 1.98)
Depressive symptoms	47.42 (45.82–49.02; 9.69)	48.42 (46.63–50.20; 10.61)	48.09 (46.25–49.92; 10.90)	−0.72 (−1.25–-0.193.16)*	−0.39(−0.94–0.17; 3.28)	0.33 (0.04–0.62; 1.71)
Fatigue	45.21 (43.35–47.08; 11.27)	46.52 (44.53–48.52; 11.90)	44.79 (42.55–47.02; 13.34)	−0.98(−1.60– −0.37; 3.68)*	0.75(0.09–1.41; 3.91)**	1.73(1.33–2.14; 2.39)*
Pain interference	42.53 (41.07–43.98; 8.81)	42.78 (41.30–44.26; 8.81)	42.46 (40.91–44.01; 9.25)	−0.06(−0.65–0.53; 3.51)	0.26(−0.26–0.77; 3.07)	0.32(0.01–0.62; 1.81)**

* = *p* < .01; ** = *p* < 0.05.

### Associations Among the Forms

Table 3 provides the ICCs among all forms for each PROMIS Pediatric domain. All PROMIS Pediatric forms were highly correlated with each other, with ICCs ranging from 0.90 to 0.99.

**Table 3.** Intra-class correlations (ICCs) of PROMIS pediatric T-scores for short form (SF) and computerized-adaptive testing (CAT) assessments

		CAT–4	CAT–5/6
		*ICC*	*ICC*
Anxiety	SF–4	0.90	0.93
	CAT–4		0.98
Depressive symptoms	SF–4	0.95	0.95
	CAT–4		0.99
Fatigue	SF–4	0.95	0.95
	CAT–4		0.97
Pain interference	SF–4	0.92	0.94
	CAT–4		0.98

### Floor and Ceiling Effects

Floor and ceiling effects for each form are presented in Table 4. All forms had relatively high floor effects that represent absence of or very mild symptom experiences. The CAT-5/6 had the lowest percentages of floor effects relative to the other two forms and the SF-4 and CAT-4 had similar percentages. All forms had relatively negligible ceiling effects.

**Table 4.** Floor and ceiling effects for PROMIS pediatric short form (SF) and computerized-adaptive testing (CAT) assessments

		Floor effects (reflecting no / very mild symptoms)			Ceiling effects (reflecting severe symptoms)		
		SF–4	CAT–4	CAT–5/6	SF–4	CAT–4	CAT–5/6
	*n*	*n* (%)	*n* (%)	*n* (%)	*n* (%)	*n* (%)	*n* (%)
Anxiety	143	35 (24%)	42 (29%)	33 (24%)	1 (1%)	0 (0%)	0 (0%)
Depressive symptoms	140	44 (31%)	41 (29%)	15 (11%)	2 (1%)	2 (1%)	2 (1%)
Fatigue	139	41 (30%)	37 (27%)	26 (19%)	3 (2%)	1 (1%)	1 (1%)
Pain interference	139	70 (50%)	73 (53%)	65 (47%)	0 (0%)	1 (1%)	0 (0%)

### Precision of Each Form

**Figure 1.**
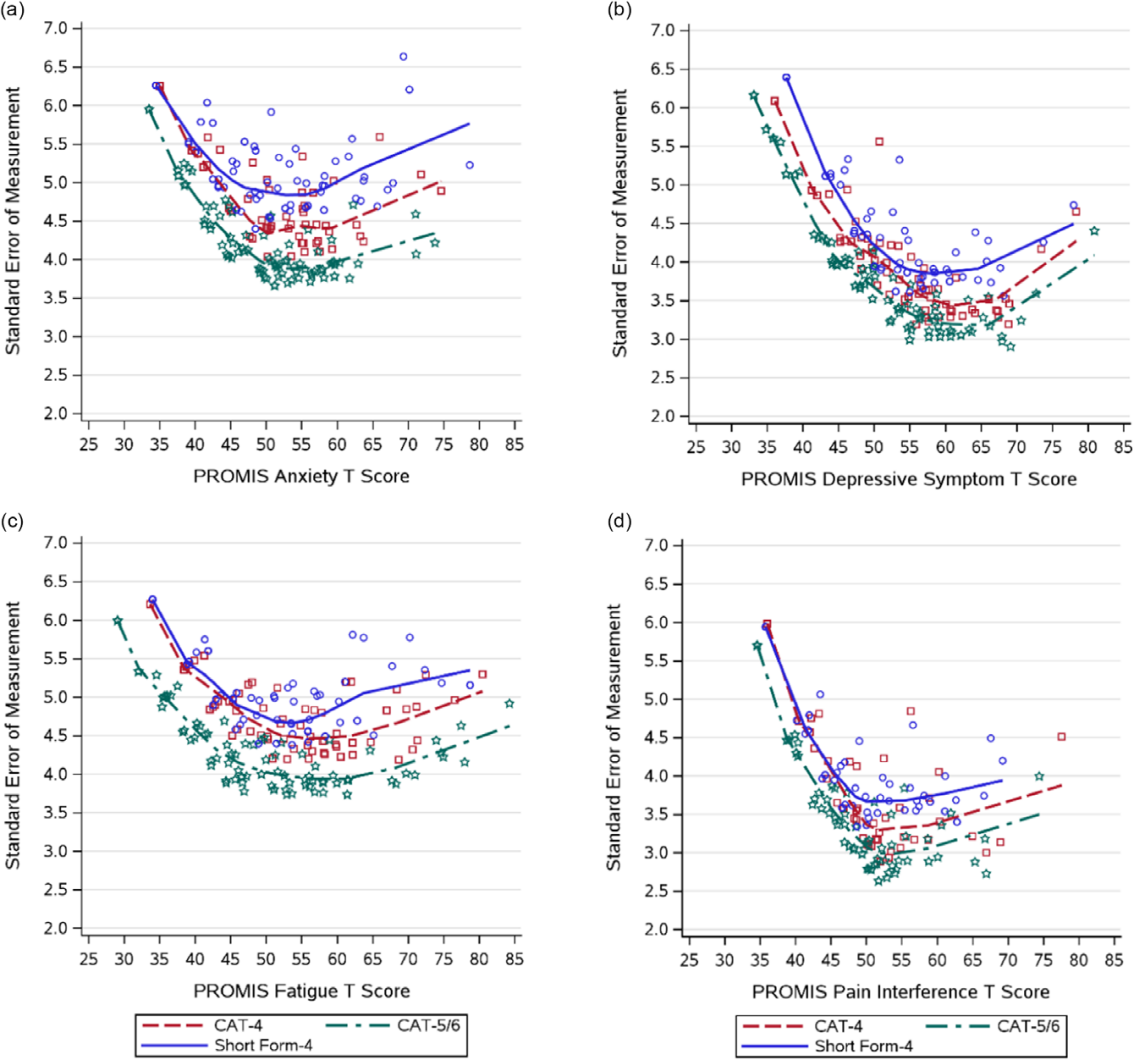
Standard error of measurement across PROMIS pediatric domain T scores for short forms (SF) versus computerized adaptive testing (CAT) assessments. a. PROMIS pediatric anxiety; b. PROMIS pediatric depressive symptoms; c. PROMIS pediatric fatigue; d. PROMIS pediatric pain interference. the CAT algorithm stopping rules are every child completes between 5 and 6 items per PROMIS pediatric scale and the CAT system stops at 5 items if the standardized standard error of measurement is < 0.40, which is 4 points on the PROMIS T-score metric (standard deviation of 10).

We compared the precision of each form by comparing the SEMs. Fig. 1 shows the SEM plots for each of the PROMIS Pediatric symptom measures. Consistently, the CAT-5/6 had the lowest SEMs across the PROMIS T-score range. In general, the CAT-4 had lower SEMs relative to the SF-4.

The average SEMs for each form are presented in Supplementary Table 3. We found statistically significant omnibus ANOVA tests for all four PROMIS domains (*p* < .01). For anxiety, depressive symptoms, and fatigue, pairwise comparisons supported the trend that the CAT-5/6 had statistically significant lower SEMs than both CAT-4 and SF-4 and the CAT-4 had a statistically significant lower SEM than the SF-4 (*p* < .01). For pain interference, we found statistically significant lower SEMs for the CAT-5/6 compared with the SF-4 and for the CAT-4 compared with the SF-4 (*p* < .01), but we did not find statistically significant differences between the two CAT versions (*p* = 0.46).

**Figure 2.**
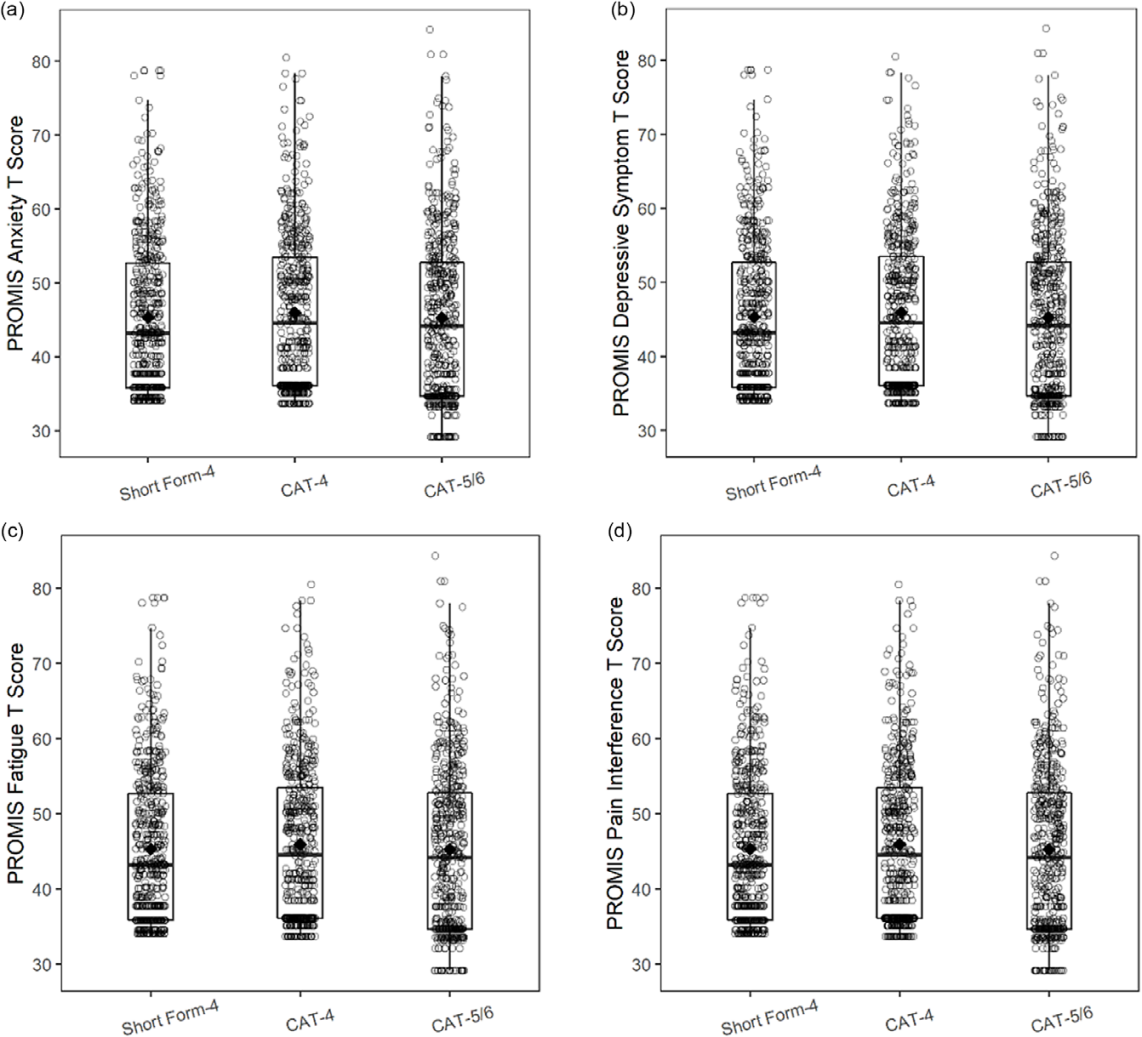
PROMIS pediatric domain T score distributions for short form (SF) versus computerized adaptive testing (CAT) administration. a. PROMIS pediatric anxiety; b. PROMIS pediatric depressive symptoms; c. PROMIS pediatric fatigue; d. PROMIS pediatric pain interference.

Fig. 2 shows the distribution of PROMIS Pediatric T scores for CAT administration versus SF administration. For all PROMIS Pediatric domains, a wider T score range was seen with the CAT-5/6 than with either the CA-4 or the SF.

### CAT and SF Ability to Differentiate Into Clinically Relevant Groups

Table 5 presents the PROMIS Pediatric symptom T-score means for the active disease and remission subgroups by form. All forms showed a statistically significant difference between active disease and remission for all PROMIS Pediatric symptom domains (i.e. the 95% CIs did not include 0). However, we found no statistically significant differences between the effect sizes when comparing the different forms (i.e. their 95% CIs included the effect size of other forms).

**Table 5.** Comparison of standardized differences between PROMIS pediatric domain mean scores among participants with remission versus active inflammatory bowel disease with use of short forms (SFs) versus computerized adaptive testing (CAT)^a^

		Patients with active disease (n = 18)	Patients in remission (n = 89)	
Domain	Form	mean (95% CI; SD)	mean (95% CI; SD)	Standardized difference (95% CI)
Anxiety	SF-4	51.35 (46.45–56.26; 9.87)	44.63 (42.57–46.69; 9.78)	−0.687 (−1.080, −0.293)
	CAT-4	53.63 (47.97–59.29; 11.37)	43.92 (41.97–45.87; 9.16)	−1.016 (−1.427, −0.606)
	CAT-5/6	52.86 (47.77–57.94; 10.23)	43.75 (41.67–45.83; 9.60)	−0.938 (−1.351, −0.525)
Depressive symptoms	SF-4	52.94 (47.54–58.35; 10.86)	45.67 (43.82–47.52; 8.79)	−0.794 (−1.191, −0.397)
	CAT-4	53.57 (47.92–59.23; 11.37)	46.61 (44.46–48.76; 9.97)	−0.681 (−1.083, −0.280)
	CAT-5/6	53.63 (47.96–59.29; 11.40)	46.04 (43.79–48.28; 10.41)	−0.717 (−1.120, −0.315)
Fatigue	SF-4	58.01 (50.93–65.10; 14.25)	42.26 (40.33–44.18; 9.16)	−1.551 (−1.988, −1.115)
	CAT-4	57.55 (50.12–64.99; 14.95)	44.00 (41.75–46.26; 10.44)	−1.197 (−1.620, −0.773)
	CAT-5/6	57.52 (48.86–66.19; 17.43)	41.92 (39.41–44.42; 11.61)	−1.221 (−1.646, −0.796)
Pain interference	SF-4	50.97 (46.02–55.91; 9.95)	40.71 (39.06–42.35; 7.83)	−1.250 (−1.668, −0.832)
	CAT-4	52.18 (47.06–57.30; 10.29)	40.53 (38.97–42.08; 7.21)	−1.492 (−1.933, −1.051)
	CAT-5/6	52.49 (47.77–57.21; 9.49)	39.98 (38.29–41.67; 7.84)	−1.536 (−1.980, −1.092)

a Absolute differences based on pooled standard deviations.

## Discussion

We compared the performance of CAT and static SF administration of the PROMIS Pediatric symptom measures in a cohort of children with IBD. Our results demonstrate that the CAT and SF PROMIS Pediatric average T-scores are similar for anxiety and pain interference, with small differences for depressive symptoms and fatigue that were not considered meaningful (<3 points). The correlation among all forms was very high (ICCs ≥ 0.90). We found that the CAT-5/6, CAT-4, and SF-4 all had relatively high floor effects representing the absence of or very mild symptoms, but that the CAT-5/6 had the least floor effects compared to the other forms. We found negligible ceiling effects across all three forms. In general, the CAT-5/6 had lower SEMs than the CAT-4 and the SF-4, and the CAT-4 had lower SEM than the SF-4, suggesting that the CAT forms offer more precision than the SF. As expected, we did not find notable differences when looking at effect size differences between IBD children with active versus quiescent disease activity.

In the present study with a cohort wherein most children did *not* have severe symptom burden, the CAT and SF performed similarly, particularly in the distribution of symptom levels that represent mild to moderate symptom burden. If a sample distribution is expected to have severe symptom burden (i.e. a clinical trial enrolling patients with moderate-severe IBD), with more participants at the extremes of measure, then researchers may prefer the improved precision and reliability of the CAT over the SF. Additionally, the lower SEM and thus higher reliability of the CAT compared to the SF means that a smaller sample size is needed to obtain a desired level of statistical power (as the SEM is inversely related to reliability). As such, the CAT may provide particular value for rare disease research or studies involving difficult and/or costly patient recruitment.

The CAT and SF instruments have been previously evaluated in the PROMIS Pediatric measures, but in a different cohort of 67 pediatric patients with juvenile myositis [29]. The present study confirms many of this study’s findings and offers additional key insights. As in our study, Patel *et al.* found that the CAT and SF had similar scores and correlated highly, and that the CAT had fewer floor effects than the SF. One important difference between the studies is that our CAT stopping rule was earlier than the Patel *et al.* study, such that participants in the present study could receive a maximum of 6 CAT questions, while those in the latter study could receive up to 12 CAT questions. Patel *et al.* recommended a follow-up study such as ours to better optimize stopping rules [29]. We showed that CAT offers improvement in floor effects and precision over the SF even with a stopping rule at six questions, which carries the added benefit of minimizing respondent burden.

Our results generally align with prior CAT versus SF studies of adult PROMIS measures including the Prosthetic Limb Uses Survey of Mobility (PLUS-M^TM^), and the Activity Measure for Post-Acute Care (AM-PAC) [17,22,30–34]. Our results and those of previous studies support an enhanced ability of the CAT (compared with the SF) to differentiate participants at the extremes of response and with lower SEM. A study of the PLUS-M^TM^ measure found that the CAT offered more reliable scores at higher levels of mobility (the outcome of interest) than the SF, but that the two test forms were similar in average scores [17]. Fries *et al.* similarly showed reduced SEM for the CAT over equal-length static SFs across the severity spectrum for the PROMIS physical function domain [31]. In a study of the AM-PAC, Haley *et al.* determined that the CAT resulted in greater precision than randomly selected static items, particularly for low- and high-performing adults [33].

Prior studies have found greater efficiency benefits (i.e. reduced respondent burden due to fewer items to administer) of the CAT over the SF. Amtmann *et al.* found the PLUS-M^TM^ SFs were often *longer* than the CATs, with 7 or 12 question SFs being compared to CATs that ranged between 4 and 12 items [17]. Likewise, Haley *et al.* found that AM-PAC CAT took 43% less time to complete than the SF version, but on average the CAT forms were 33% shorter than the SFs [34]. In our study, we used a very brief SF of four items; thus it would be unrealistic to observe CATs that could yield shorter assessment and meet the SEM threshold of 0.40. Both the SF-4 and the CAT-4 rarely could go under the 0.4 threshold (indicating lack of precision). Thus, the longer CAT-5/6 is desirable if reliability is a primary concern. A reliable measure would be an important criterion if the HRQOL outcome was a primary or secondary endpoint in a trial. Findings here would support use of the CAT-5/6 or a SF longer than four items.

In addition, the electronic administration of the CAT and associated software costs must be weighed against the potential benefits of the CAT over the SF for a particular study population. If the study includes participants who are expected to be at the severe end of symptom burden and the study is funded sufficiently to support the added costs for CAT, then the CAT becomes a better option. Conversely, if researchers anticipate that most respondents will not have a high symptom burden and they desire to have both electronic and paper forms, then the SF may be preferred.

Strengths of this study include the use of a geographically diverse cohort of children with IBD. As a chronic, relapsing-remitting condition in which strong performance of the PROMIS measures is already well-established [7–9], pediatric IBD represents an ideal population in which to compare the use of CAT versus static SFs. The study was designed such that children concurrently completed both forms of the CAT along with the static SF, facilitating direct, head-to-head comparison. The self-report nature of IBD Partners ensured that collected data came directly from children and adolescents, which allowed for accurate assessment of PRO testing modalities. Regarding study limitations, the overall low symptom burden of the cohort limited our ability to evaluate the CAT and SF performance at extremes of the distribution. Another limitation is that there was considerable overlap between the items included in the CAT and the SF, as both forms were selecting the most discriminating items. Future studies, including use of simulation comparing longitudinal data, should focus on using different items in each form for comparison. Additional limitations include the volunteer nature of the cohort. Most participants were white and non-Hispanic, which may limit generalizability to other populations. IBD status was also self-reported instead of physician-confirmed, although it is reassuring that a validation study performed in the adult IBD Partners cohort showed high reliability of self-reported IBD status [35]. Finally, IBD Partners includes only English-speaking patients and does not collect data on literacy. Future studies investigating the performance of CAT vs SFs in populations who cannot read or for whom English is a second language would be of value. The PROMIS measures have been translated into a wide variety of languages including French, German, and Spanish. The website HealthMeasures.net provides a list of current translations that vary for each PROMIS measure [36].

We found similar average scores for the CAT versus SF administration of the PROMIS Pediatric domains to children and adolescents with IBD but determined that the CAT resulted in increased precision and fewer floor effects than the SF. Researchers seeking to incorporate the PROMIS Pediatric measures into their trials should consider CAT if they anticipate that their sample will skew toward the extremes of the symptom severity spectrum or if a smaller sample size is anticipated. Otherwise, the benefits of the CAT over the traditional static SF may not outweigh the added cost and computer-based administration requirement.

## Supplementary material

For supplementary material accompanying this paper visit https://doi.org/10.1017/cts.2023.526. click here to view supplementary material
