# Supplementary material for: Computerized-adaptive testing versus short forms for pediatric inflammatory bowel disease patient-reported outcome assessment
Source: J Clin Transl Sci. 2023 Apr 14;7(1):e109. doi: 10.1017/cts.2023.526 (PMC10225267; doi:10.1017/cts.2023.526)
Supplement: Supplementary file 1 [file S2059866123005265sup001.docx]

Supplementary Table 1. Minimum and maximum scores for computerized adaptive testing (CAT) and static short forms (SFs) by domain

| PROMIS Pediatric Domain | SF-4 | | CAT-4 | | CAT-5/6 | |
| --- | --- | --- | --- | --- | --- | --- |
|  | Minimum | Maximum | Minimum | Maximum | Minimum | Maximum |
| Anxiety | 34.5 | 78.7 | 35.0 | 83.3* | 33.5 | 83.3* |
| Depressive symptoms | 37.7 | 78.0 | 36.1 | 78.4 | 33.1 | 80.9 |
| Fatigue | 34.0 | 78.7 | 33.7 | 80.5 | 29.1 | 84.3 |
| Pain interference | 35.8 | 73.5 | 36.0 | 77.6 | 34.6 | 78.0* |

*For scores noted with an asterisk, no respondent in the data set achieved the maximum score for this domain and form type, so the reported score maximums were taken from the National Institutes of Health Patient Reported Outcomes Measurement Information System^®^ manual scoring tables.

Supplementary Table 2. Amount of overlap between computerized adaptive testing (CAT) and static short forms (SFs) in study cohort

| PROMIS Pediatric Domain | Number of SF items that overlap (N [%])* | | | | | | | | | |
| --- | --- | --- | --- | --- | --- | --- | --- | --- | --- | --- |
|  | CAT-4 | | | | | CAT 5/6 | | | | |
|  | 0 | 1 | 2 | 3 | 4 | 0 | 1 | 2 | 3 | 4 |
| Anxiety | 0 (0%) | 7 (5%) | 60 (42%) | 76 (53%) | 0 (0%) | 0 (0%) | 2 (1%) | 53 (37%) | 88 (62%) | 0 (0%) |
| Depressive symptoms | 0 (0%) | 46 (33%) | 65 (46%) | 29 (21%) | 0 (0%) | 0 (31%) | 43 (40%) | 46 (29%) | 41 (0%) | 0 (0%) |
| Fatigue | 0 (0%) | 0 (0%) | 128 (92%) | 11 (8%) | 0 (0%) | 0 (0%) | 0 (0%) | 87 (63%) | 47 (34%) | 5 (4%) |
| Pain interference | 1 (1%) | 16 (12%) | 27 (20%) | 77 (56%) | 17 (12%) | 1 (1%) | 15 (11%) | 28 (20%) | 3 (2%) | 91 (66%) |

*The number and proportion of SF items that are identical to those on the CAT form completed by a single respondent within the study cohort.

Supplementary Table 3. Mean standard error of measurement for PROMIS Pediatric short form (SF) and computerized adaptive testing (CAT) assessments

| PROMIS Pediatric Domain | SF-4 | CAT-4 | CAT-5/6 |
| --- | --- | --- | --- |
|  | mean (SD) | mean (SD) | mean (SD) |
| Anxiety | 5.37 (0.64) | 5.07 (1.03) | 4.47 (1.19) |
| Depressive symptoms | 5.04 (1.09) | 4.55 (1.14) | 4.19 (1.09) |
| Fatigue | 5.40 (0.68) | 5.18 (0.73) | 4.68 (0.78) |
| Pain interference | 5.00 (1.04) | 4.91 (1.23) | 4.49 (1.22) |
